# Structural Conservation and Transcriptional Plasticity of *atp2a1* in *Acrossocheilus fasciatus* Under Temperature and Flow Acclimation

**DOI:** 10.3390/genes16111385

**Published:** 2025-11-15

**Authors:** Ye Chen, Yongyao Guo, Peihao Cai, Zhangjie Chu, Bo Zhao

**Affiliations:** 1 Key Laboratory of Sustainable Utilization of Technology Research for Fisheries Resources of Zhejiang Province, Zhejiang Marine Fisheries Research Institute, Zhoushan 316000, China; jimmycy85@163.com (Y.C.); g353746433@gmail.com (Y.G.); 2 College of Fisheries, Zhejiang Ocean University, Zhoushan 316000, China; caipeihao1015@zjou.edu.cn (P.C.); czj0501@zjou.edu.cn (Z.C.)

**Keywords:** *Acrossocheilus fasciatus*, *atp2a1*, thermal and flow acclimation, muscle adaptation

## Abstract

Background/Objectives: The sarcoplasmic reticulum Ca^2+^-ATPase 1 (Atp2a1) is a key regulator of calcium homeostasis and muscle relaxation, yet its roles in fish remain poorly understood. Methods: We investigated the structural characteristics, phylogenetic relationships, and transcriptional regulation of *atp2a1* in *Acrossocheilus fasciatus*, a stream-dwelling cyprinid sensitive to environmental fluctuations. Results: Bioinformatic analyses revealed that the 991-aa Atp2a1 protein is highly conserved among teleosts but exhibits divergence from mammals in the Cation_ATPase_N domain and transmembrane regions TM3, TM9, and TM10. Phylogenetic analysis clustered *A. fasciatus* most closely with *Onychostoma macrolepis*. Tissue-specific qRT-PCR demonstrated predominant expression in skeletal muscle, followed by testis, brain, heart, and gill. Promoter prediction identified binding motifs for KLF9, CTCF, MAZ, KLF5, ONECUT3, and HOXB13. qRT-PCR analysis showed that long-term cold acclimation (16 °C vs. 24 °C) markedly downregulated *atp2a1* expression (ANOVA, *p* < 0.05, *n* = 3), whereas moderate flow velocity (2 BL·s^−1^ vs. 0 BL·s^−1^) significantly upregulated it (ANOVA, *p* < 0.05, *n* = 3). Alternative splicing analysis based on RNA-seq data further revealed a corresponding decrease and increase in skipped exon (SE) inclusion under cold and flow conditions, respectively (*P*_adj_ < 0.05). Conclusions: These results further raise the possibility that the regulatory complexity of *atp2a1* contributes to adaptation of teleosts under fluctuating environments.

## 1. Introduction

The Sarcoplasmic Reticulum Ca^2+^-ATPase 1 (Atp2a1) is a P2-type ATPase that drives Ca^2+^ re-uptake into the sarcoplasmic reticulum and is a key determinant of muscle relaxation [1,2]. In zebrafish, *atp2a1* is highly expressed in embryonic skeletal muscle, and its loss causes the accordion phenotype with sustained contractions, underscoring its role in Ca^2+^ homeostasis and muscle relaxation [3]. Moreover, the zebrafish Atp2a1 sequence and domain organization are highly conserved across vertebrates, further supporting its fundamental role in Ca^2+^ regulation [4]. These functional domains align with the canonical ten-helix transmembrane core and cytosolic A/P/N domains defined by ATP2A1 crystal structures, providing a structural framework widely extrapolated to teleost Atp2a1 [5]. As a central player in muscle energy metabolism and Ca^2+^ signaling, the biological functions of the ATP2A1 gene have been extensively characterized in mammals [6,7,8]. However, there remains a significant knowledge gap regarding its roles in fish.

Despite this gap, the few available studies suggest that *atp2a1* transcription in fish is plastic under environmental stressors, particularly thermal challenges. In Japanese medaka (*Oryzias latipes*), acute cold challenge significantly elevates *atp2a1* (together with *sln*) in both red and white skeletal muscle [9]. In regionally endothermic scombrids such as Pacific bluefin tuna (*Thunnus orientalis*) and swordfish (*Xiphias gladius*), quantitative PCR analyses have shown that *atp2a1* transcript levels are several-fold higher in heater organ and oxidative red muscle compared with white muscle and with phylogenetically related ectothermic species such as mackerel [10]. Beyond these cases, zebrafish exhibit temperature-responsive regulation of *atp2a1*/*atp2a1l* transcripts, reinforcing the view that Atp2a1-mediated Ca^2+^ handling is thermally modulated in fish [11]. Collectively, these studies indicate that *atp2a1* transcription in fish is responsive to environmental stressors; however, detailed mechanistic investigations in fish skeletal muscle remain scarce. Moreover, while mammalian work demonstrates that alternative splicing of ATP2A1 can influence myogenesis and Ca^2+^ handling [12,13], comparable splice-functional studies in fish are virtually absent. Together, these findings highlight *atp2a1* as an important regulator of muscle calcium dynamics in fish, while also underscoring the need for further studies to elucidate its regulatory mechanisms.

*Acrossocheilus fasciatus* is an economically important freshwater fish species widely distributed in Zhejiang Province [14]. In its native habitats, it experiences pronounced seasonal changes in water temperature and flow velocity. Moreover, evidence shows that this species rapidly responds to environmental fluctuations, suggesting its potential as an ecological indicator [15,16,17]. In particular, its high temperature sensitivity provides a basis for exploring the molecular mechanisms underlying thermal responses [15,18,19]. Furthermore, its genome has recently been published [20,21], and we have also obtained and analyzed full-length transcriptomic data for this species [18]. Analyses of full-length transcriptomic data revealed that *atp2a1* (AFCHR12_02800.1) displays extensive alternative splicing and polyadenylation site modifications (Figure S1A–G), suggesting that further investigation of *atp2a1* may yield new insights into its regulatory complexity and functional roles. In this study, we performed bioinformatic analyses of *atp2a1* based on genomic and transcriptomic resources. We further validated its tissue-specific expression using qRT-PCR, and mined our laboratory's available transcriptome datasets to examine *atp2a1* expression under temperature, and flow velocity stress conditions. These analyses provide a molecular framework for future functional studies of *atp2a1* in *A. fasciatus*.

## 2. Materials and Methods

### 2.1. Research Background

Two healthy adult *A. fasciatus* (one male and one female; average body weight 13.03 ± 1.01 g, ~1 year old) were obtained from a commercial aquaculture farm and temporarily maintained at Zhejiang Ocean University under controlled temperature (24 ± 1 °C) and photoperiod (12 h light:12 h dark) conditions. The fish were then used for full-length transcriptome sequencing (CRA020749) [18], in which 11 tissues—including the brain, gills, heart, spleen, head kidney, liver, intestine, testes, skin, dorsal muscle, and ovaries—were sampled (Table S1). The third-generation (PacBio) sequencing and data processing were entirely performed by Sangon Biotech Co., Ltd. (Shanghai, China) (https://www.sangon.com/; accessed on 15 August 2025). Through an in-depth analysis of this dataset 8968 exhibited more than one splicing isoform (Figure S1A). KEGG pathway analysis revealed these genes were primarily associated with Human Diseases, Cellular Processes, and Environmental Information Processing (Figure S1B). Genes with the highest frequency of exon skipping included *actin*, *rad21*, *plk2*, *dcx*, *ccna2*, and *atp2a1* (Figure S1C). Polyadenylation (polyA) sites were detected in 10,755 genes, with 3743 genes exhibiting variable polyadenylation sites (Figure S1D). KEGG analysis further demonstrated that these genes were mainly involved in Human Diseases, Cellular Processes, Environmental Information Processing, and Organismal Systems (Figure S1E). Genes with the highest number of polyA sites included *atp2a1* and *mhy* (Figure S1F). Notably, *atp2a1* (AFCHR12_02800.1) ranked among the top 10% genes in both the number of splice isoforms and polyA sites. Further investigation revealed that *atp2a1* possesses 781 isoforms, 3353 alternative splicing events, and eight distinct polyA sites (Figure S1G). Currently, the most widely acknowledged function of ATP2A1 is its role in regulating non-shivering thermogenesis. However, the extensive post-transcriptional modifications observed across multiple tissues indicate that *atp2a1* may possess additional, as yet unexplored, biological functions, thus necessitating further investigation.

### 2.2. Bioinformatic Analysis of the atp2a1 Gene

Based on the publicly available genome (GCA_039880705.1), the *atp2a1* gene sequence (AFCHR12_02800.1) was retrieved, and the corresponding amino acid sequence (Supplementary Material S1) was predicted using ORFfinder (https://www.ncbi.nlm.nih.gov/orffinder/; accessed on 15 August 2025). Physicochemical properties were analyzed with ProtParam (https://web.expasy.org/protparam/; accessed on 15 August 2025) [22], and the hydrophilicity and hydrophobicity of the sequence were analyzed using ProtScale (https://web.expasy.org/protscale/; accessed on 15 August 2025) [22]. Subcellular localization was analyzed with DeepLoc v2.0 (https://services.healthtech.dtu.dk/services/DeepLoc-2.0/; accessed on 20 October 2025) [23]. Domain prediction was performed using the SMART v10.0 (https://smart.embl.de/; accessed on 15 August 2025) [24]. The transmembrane regions (TMRs) were predicted using DeepTMHMM v1.0.42 (https://dtu.biolib.com/DeepTMHMM/; accessed on 15 August 2025) [25], while signal peptides were predicted using SignalP v6.0 Server (https://services.healthtech.dtu.dk/services/SignalP-6.0/; accessed on 15 August 2025) [26]. Protein secondary structure prediction was conducted with Psipred v4.0 (http://bioinf.cs.ucl.ac.uk/psipred/; accessed on 15 August 2025) [27], and the tertiary structure was retrieved from the Swissmodel (https://swissmodel.expasy.org/; accessed on 15 August 2025) [28]. The model with the highest GMQE (0.81) and QMEANDisCo global score (0.78 ± 0.05) was selected. Template PDB 5xa7.1.A, corresponding to the sarcoplasmic/endoplasmic reticulum calcium ATPase 1A (ATP2A1) with 83.75% sequence identity, was used for homology modeling (Supplementary Material S2). The same procedure was applied to *Onychostoma macrolepis*, yielding a model with a GMQE of 0.80 based on the same template (Supplementary Material S3). All analyses were conducted under default parameters. For comparison of the three-dimensional structures, the AlphaFold Protein Structure Database (https://alphafold.ebi.ac.uk/, release of March 2025; accessed on 15 August 2025) was utilized [29]. The predicted structures of *Danio rerio* Atp2a1 (AF-Q642Z0-F1-v6; average pLDDT = 88.12, high confidence) and *Homo sapiens* ATP2A1 (AF-O14983-2-F1-v6; average pLDDT = 89.12, high confidence) were retrieved. Structural alignment was performed using PyMOL v3.0.3, comparing the predicted *A. fasciatus* Atp2a1 structure with those of *D. rerio, O. macrolepis* and *H. sapiens*, and the root mean square deviation (RMSD) was calculated to assess structural similarity.

Homologous Atp2a1 protein sequences were obtained from the NCBI database through BLAST (https://blast.ncbi.nlm.nih.gov/Blast.cgi; accessed on 15 August 2025), and a phylogenetic tree was constructed using the maximum likelihood (ML) method with the JTT+G+I model and 1000 bootstrap replicates in MEGA v7.0 [30]. Multiple sequence alignment of Atp2a1 from different fish species was performed using Clustal Omega (https://www.ebi.ac.uk/Tools/msa/clustalo/; accessed on 15 August 2025) [31].

### 2.3. atp2a1 Gene Tissue Expression Analysis

To investigate the tissue-specific expression of the *atp2a1* gene in *A. fasciatus*, total RNA was extracted from a variety of tissues, including the brain, gill, heart, spleen, head kidney, liver, pancreas, intestine, skin, dorsal muscle, testis, and ovary (Table S1). RNA extraction was performed using the TRIzol reagent, and RNA quality and concentration were assessed using Nanodrop and Agilent 2100 Bioanalyzer. RNA integrity was confirmed by agarose gel electrophoresis. Differential expression of *atp2a1* across these tissues was analyzed using quantitative reverse transcription qRT-PCR. First, cDNA was synthesized from total RNA using the PrimeScript RT reagent Kit (RR047A; Takara Bio Inc., Shiga, Japan) according to the manufacturer’s protocol. qPCR was performed on a QuantStudio^TM^ Real-Time PCR System (Thermo Fisher Scientific, Waltham, MA, USA). Each 25 μL reaction contained 12.5 μL of TB Green Premix Ex Taq (Tli RNaseH Plus, 2×) (AG11719; ACCURATE BIOTECHNOLOGY(HUNAN)CO.,LTD, ChangSha, China), 2.0 μL of cDNA template, 0.5 μL of each forward and reverse primer, and 9.5 μL of nuclease-free water. The amplification program was as follows: initial denaturation at 95 °C for 30 s, followed by 40 cycles of 95 °C for 5 s and 60 °C for 30 s. Primers for *atp2a1* amplification were designed by Primer 5.0 software [32] based on the genome sequence, with *β-actin* as an internal control [33] for normalization (Table S2). The expression levels of *atp2a1* in each tissue were calculated using the 2^^−ΔΔCt^ method, with muscle tissue set as the calibrator. Statistical analysis was performed using a one-way ANOVA, followed by Duncan’s post hoc test for multiple comparisons, to identify tissues with significant differences in *atp2a1* expression levels. The results were considered statistically significant at *p* < 0.05.

### 2.4. Promoter Region Prediction of atp2a1

The 2000 bp upstream sequence from the translation start codon (ATG) of the *atp2a1* gene was retrieved and used for promoter region analysis (Supplementary Material S4). The potential transcription start site (TSS) of the *atp2a1* gene was predicted using the online tool BDGP (http://www.fruitfly.org/seq_tools/promoter.html; accessed on 15 August 2025) [34], with the minimum promoter score set to 0.8. CpG islands within the promoter region were identified using MethPrimer (http://www.urogene.org/cgi-bin/methprimer/methprimer.cgi; accessed on 15 August 2025) [35], with the following criteria: length > 100 bp, observed/expected ratio > 0.6, and GC content > 50%. Potential transcription factor binding sites (TFBS) were identified using the Fimo Binding Motif Scan module in TBtools v2.363 [36], based on the JASPAR Vertebrate PFMs (non-redundant) database (https://jaspar.elixir.no/downloads/; accessed on 20 October 2025). Sites with motif scores > 10 and q-value < 0.05 were retained as putative TFBSs, and those located within the BDGP-predicted promoter region were further analyzed and discussed.

### 2.5. Expression Profiling of atp2a1 Under Various Stress and Acclimation Conditions

We have conducted a series of comprehensive experiments on *A. fasciatus*, focusing on the outcomes of long-term acclimation. These include prolonged thermal acclimation and extended adaptation to varying flow velocities.

Long-term thermal acclimation treatments were conducted at 16 °C, 20 °C, 24 °C, and 28 °C. *A. fasciatus* with an average body weight of 0.57 ± 0.16 g were used. Experiments were performed in 15 L tanks, with the initial water temperature set at 20 °C and adjusted at a rate of 1 °C per day until the target temperature was reached. Each treatment included three replicate tanks, with 30 fish per tank exposed to identical conditions, totaling 360 individuals (4 temperature levels × 3 replicates × 30 fish). Each tank was treated as an independent biological replicate. Fish were then maintained at the target temperature for 60 days [19]. Muscle tissues were collected for transcriptome sequencing and subsequent analyses.

Long-term flow velocity acclimation treatments were conducted at three flow regimes: static water (0 BL s^−1^), moderate flow (2 BL s^−1^), and high flow (4 BL s^−1^). *A. fasciatus* with an average body weight of 1.00 ± 0.30 g were used. Experiments were performed in 127 L circular tanks (60 cm in diameter × 45 cm in height) with the water temperature maintained at 24 °C. Flow velocity was measured every 15 days to ensure stability. Starting from static conditions, the flow was increased by 1 BL s^−1^ per day until the target velocity was reached. Fish were then maintained at the designated flow conditions for 60 days. During the experiment, fish were subjected to daily swimming training for 11 h (09:00–18:00 and 19:00–21:00). Each treatment included three replicate tanks with 30 fish per tank, totaling 270 individuals (3 flow velocities × 3 replicates × 30 fish). Each tank was considered an independent unit of replication for downstream analyses. After the acclimation period, muscle tissues were collected for transcriptome sequencing and subsequent analyses.

For the long-term treatments, temperature-related transcriptomic data were retrieved from the NGDC database (CRA020752), while flow velocity-related transcriptome data were accessed from NGDC (CRA029280). Differentially expressed genes (DEGs) were identified using DESeq2 [37] in R based on raw read counts, with 24 °C and 0 BL s^−1^ as the control conditions. Multiple testing correction was performed using the Benjamini–Hochberg false discovery rate (FDR) method, and genes with *P*_adj_ < 0.05 were considered significantly differentially expressed. Gene expression levels were calculated as log_2_(FPKM) for visualization and presented as mean ± SD. qRT-PCR analysis was performed as described in Section 2.4, with three biological replicates included in each experimental group. For qRT-PCR data, variance homogeneity was first examined, followed by one-way analysis of variance (ANOVA), and significant differences among groups were determined using Duncan’s multiple range test (*p* < 0.05).

Alternative splicing (AS) events of *atp2a1* were analyzed using the rMATS software package (v4.0.2) [38], focusing on skipped exon (SE) events. Paired-end reads of 150 bp were used as input, with three biological replicates per group. Default parameters were applied, requiring a minimum of five reads supporting each junction. Significant differential SE events were identified using a false discovery rate (FDR) < 0.05.

## 3. Results

### 3.1. Bioinformatic Analysis and Phylogenetic Analysis

The physicochemical properties of the *atp2a1*-encoded protein were predicted, revealing that it consists of 991 amino acids. Valine (Val) and alanine (Ala) were the most abundant, with 92 and 89 residues, respectively, accounting for 9.3% and 9.0% of the total. Histidine (His) was the least abundant, with only 11 residues (1.1%). The protein contains 128 negatively charged amino acids (Asp+Glu) and 97 positively charged amino acids (Arg+Lys). Its relative molecular weight (MW) is 108.73 kDa, with an aliphatic index of 95.33, an estimated isoelectric point (pI) of 5.02, and an instability index of 36.27, indicating that the protein is stable. Subcellular localization analysis predicted that Atp2a1 is primarily located in the endoplasmic reticulum. Hydropathy analysis showed that leucine (Leu) at position 95 had the strongest hydrophobicity (3.744), while arginine (Arg) at position 131 had the highest hydrophilicity (−2.411). Most amino acids showed positive hydrophobicity values (Figure S2A). Domain analysis revealed the presence of one Cation_ATPase_N domain (Figure S2B). Transmembrane domain predictions revealed 10 TMRs (Figure S2C). Signal peptide analysis indicated a Signal Peptide (Sec/SPI) value of 0.000257, with an “Other” value of 0.999743, confirming that Atp2a1 is a non-signal peptide protein (Figure S2D). Secondary structure analysis showed that the protein contains 47.23% α-helices, 15.34% β-sheets, and 37.44% random coils (Figure S2E).

**Figure 1:**
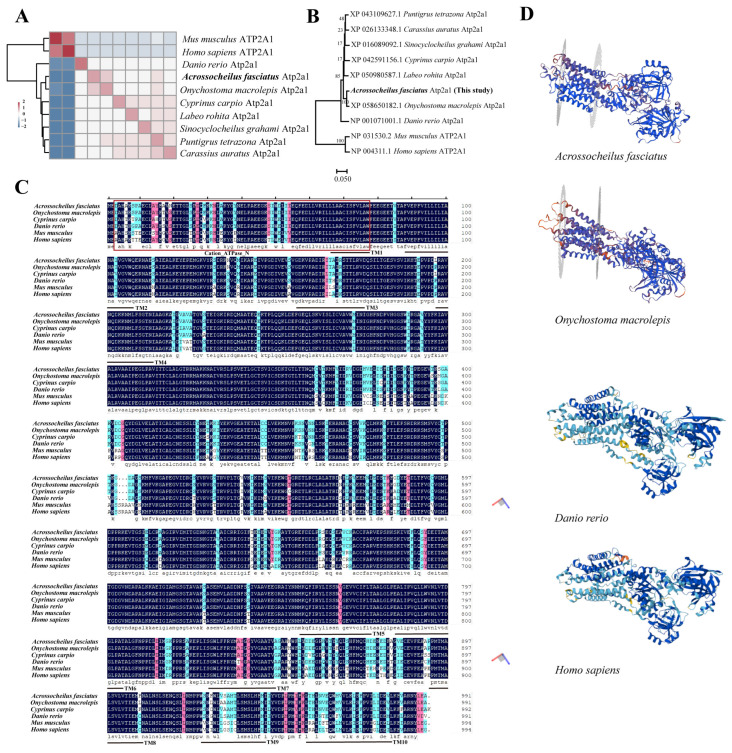
Comparative analysis of Atp2a1 among *Acrossocheilus fasciatus* and other vertebrates. (**A**) Amino acid homology analysis of Atp2a1. (**B**) Neighbor-joining phylogenetic tree based on Atp2a1 amino acid sequences. (**C**) Conserved domain analysis of Atp2a1. (**D**) Predicted 3D structures of the Atp2a1 protein in *A. fasciatus*, *O. macrolepis*, *D. rerio*, and *H. sapiens*. The structures of *A. fasciatus* and *O. macrolepis* were generated by homology modeling using SWISS-MODEL and are colored by local model quality (QMEANDisCo: blue = high, white/cyan = intermediate, yellow/orange = low). The structures of *D. rerio* and *H. sapiens* were obtained from the AlphaFold Protein Structure Database and are colored by pLDDT (blue > 90; cyan 70–90; yellow 50–70; orange < 50).

Homology analysis showed that the Atp2a1 amino acid sequence of *A. fasciatus* exhibits high similarity to other fish (98.08% with *Onychostoma macrolepis*, 95.86% with *Cyprinus carpio*, 94.05% with *Danio rerio*) and lower similarity to mammals (84.36% with both *Mus musculus* and *Homo sapiens*) (Table S3). After Z-score correction, a clear divergence between mammals and fish was evident. Moreover, as stream-dwelling cyprinids, *A. fasciatus* and *O. macrolepis* clustered together, separating from other cyprinid fish (Figure 1A). A neighbor-joining phylogenetic tree constructed from sequences of multiple fishes and mammals further clustered *A. fasciatus* most closely with *O. macrolepis* (Figure 1B). Sequence alignment highlighted pronounced variation between mammals and fish in the Cation_ATPase_N domain and TM3, TM9, and TM10 regions (Figure 1C). Consistently, tertiary structure prediction showed that *A. fasciatus* Atp2a1 shares a highly conserved overall fold with *O. macrolepis*, with only local differences relative to the *D. rerio* and *H. sapiens* ATP2A1 structures (Figure 1D). Structural alignment revealed RMSD values of 0.080 Å between *A. fasciatus* and *O. macrolepis*, 11.149 Å between *A. fasciatus* and *D. rerio*, and 10.969 Å between *A. fasciatus* and *H. sapiens*.

### 3.2. Expression of A. fasciatus atp2a1 Under Different Tissues

**Figure 2:**
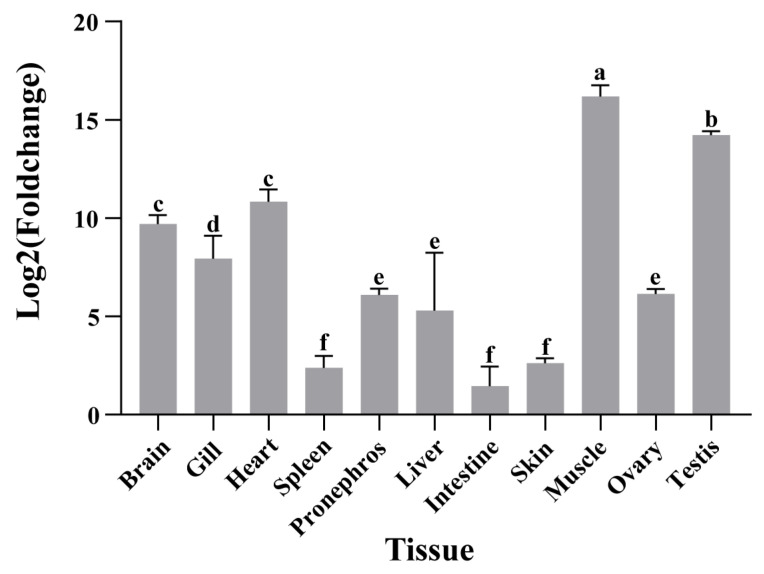
Tissue-specific expression of *atp2a1* in *Acrossocheilus fasciatus*. Data represent mean ± SD (n = 3). Gene expression levels were normalized to *β-actin*. Statistical analysis was performed using one-way ANOVA followed by Duncan’s multiple range test (*p* < 0.05). Different letters above the bars indicate significant differences among groups.

qRT-PCR analysis of multiple tissues in *A. fasciatus*, including the brain, gill, heart, spleen, head kidney, liver, pancreas, intestine, testis, skin, dorsal muscle, and ovary, showed that *atp2a1* expression was highest in dorsal muscle, followed by the testis, and then by the brain, heart, and gill (*p* < 0.05, n = 3; Figure 2).

### 3.3. Promoter Prediction

Prediction of potential TSSs for the *atp2a1* gene in *A. fasciatus* revealed five candidate sites within the promoter region, each with a prediction score of at least 0.82. These sites were located at positions 201 to 251, 255 to 305, 1038 to 1088, 1480 to 1530, and 1921 to 1971 (Supplementary Material S4; Table 1). CpG island analysis indicated the absence of CpG islands in the promoter region (Figure S3). Furthermore, prediction of TFBSs for the atp2a1 promoter identified 25 transcription factors (Supplementary Material S5). Among them, binding sites for KLF9 (216–228) were located within the 201–251 promoter region, whereas CTCF (1485–1517), MAZ (1498–1505), and KLF5 (1497–1506) were located within the 1480–1530 region. In addition, ONECUT3 (1924–1935) and HOXB13 (1929–1937) sites were positioned within the 1921–1971 region, suggesting potential regulatory roles of these transcription factors in *atp2a1* expression (Table 2).

### 3.4. Regulation of A. fasciatus atp2a1 Expression and SE Alternative Splicing Under Temperature and Flow Velocity Treatments

**Figure 3:**
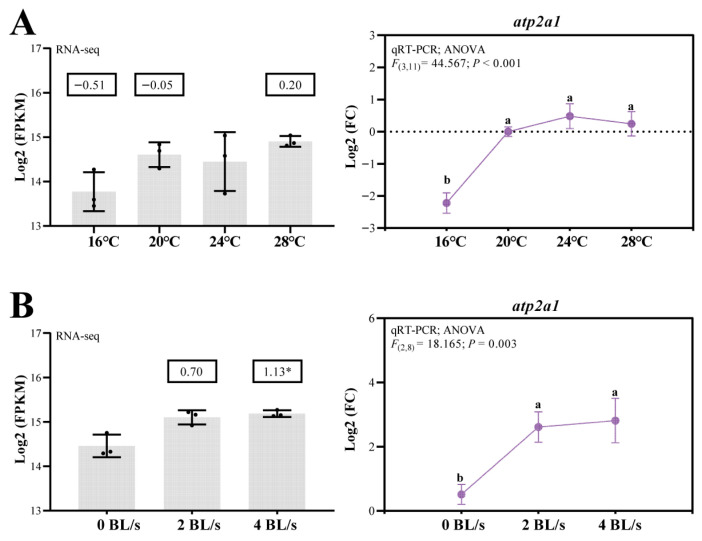
Expression of *atp2a1* under different treatments as measured by RNA-seq and qRT-PCR. (**A**) Different temperatures. (**B**) Different flow velocities. Log2FC values from RNA-seq are shown within the boxes. For RNA-seq data, asterisks indicate significant differences (*P*_adj_ < 0.05). For qRT-PCR results, statistical analysis was performed using Duncan’s multiple range test, and different letters denote significant differences among groups (*p* < 0.05). Values are presented as mean ± SD.

Further analysis of the transcriptomic and qRT-PCR data revealed distinct temperature- and flow velocity-responsive mechanisms for the expression of the *atp2a1* gene in muscle. During long-term acclimation to temperature, exposure to lower temperatures (16 °C) resulted in a significant decrease in *atp2a1* expression, while no differences were observed at 20 °C, 24 °C, and 28 °C (Figure 3A). Moreover, under long-term flow velocity treatment, flow velocity stimulation significantly promoted the expression of *atp2a1* (Figure 3B). Notably, differential skipped exon (SE) alternative splicing events were detected in the 16 °C vs. 24 °C and 2BL s^−1^ vs. 0BL s^−1^ comparisons. The corresponding PSI values were −0.131 and 0.028, respectively, indicating that SE inclusion of *atp2a1* decreased under 16 °C, whereas it increased under 2BL s^−1^ flow velocity (Supplementary Material S6).

## 4. Discussion

The *atp2a1* gene, a crucial regulator of calcium homeostasis and muscle relaxation, has been extensively characterized in mammals and validated in a limited number of fish species. In mammalian models, overexpression of ATP2A1 restores calcium homeostasis and ameliorates muscular dystrophy pathology, while reduced ATP2A1 function is linked to contractile dysfunction and age-related muscle weakness [8]. Based on previous reports, in fish, *atp2a1* has been implicated in thermogenic responses to cold exposure, where enhanced Atp2a1 activity contributes to heat production and thermal compensation mechanisms [9,10]. However, our long-term acclimation experiments under temperature and flow velocity treatments provide new insights beyond previous reports. In *A. fasciatus*, we observed that *atp2a1* expression decreased under chronic cold exposure, accompanied by a reduction in alternative splicing events, which corresponded to growth retardation [14]. In contrast, under moderate flow stimulation (2 BL·s^−1^), both *atp2a1* expression and splicing diversity were significantly enhanced, paralleling accelerated growth performance [39]. Together with our full-length transcriptome evidence revealing extensive alternative splicing and polyadenylation site usage of *atp2a1*, as well as the potential structural differences in Atp2a1 among stream-dwelling fishes, zebrafish, and humans, these findings highlight that the complex regulatory architecture of this gene contributes to its well-established roles in calcium regulation and thermal adaptation and may also be involved in growth and developmental processes.

### 4.1. Phylogenetic Conservation, Structural Divergence of atp2a1

Homology and phylogenetic analyses showed that the Atp2a1 amino acid sequence in *A. fasciatus* is highly conserved among teleosts, such as *O. macrolepis* and *C. carpio*, and also retains substantial similarity to mammalian ATP2A1 (>83%). Nevertheless, clear divergence is evident between fish and mammals, and even within cyprinids, stream-dwelling species such as *A. fasciatus* and *O. macrolepis* cluster separately from common carp, reflecting differences in their ecological habitats and physiological characteristics. The most pronounced differences are localized to the Cation_ATPase_N region and specific transmembrane segments (TM3, TM9, and TM10). Structural studies of mammalian ATP2A1a indicate that catalytic and ion-coordinating residues are highly conserved, whereas peripheral residues are more tolerant to substitutions [8]. However, our results revealed marked differences between fish and mammals in the Cation_ATPase_N region, which likely reflect distinct physiological demands and muscle calcium-handling requirements between ectothermic and endothermic vertebrates. Mutations in this domain may disrupt the coupling between ATP binding and head-domain movements, thereby reducing catalytic efficiency without necessarily abolishing ion transport. Analysis of mutations in the N-domain loop region demonstrated that disruption of this interface decreased the ATPase turnover rate by approximately 60%, while Ca^2+^ affinity remained largely unchanged [40]. These findings suggest that structural variations modify the efficiency of energy transduction rather than eliminating binding sites or Ca^2+^ recruitment. The transmembrane domain of ATP2A can be broadly divided into two regions: (a) a mobile region formed by TM1–TM6, which plays a central role in controlling access to the Ca^2+^ binding sites and contains the energy-transduction domain, a network of sarcoplasmic reticulum residues essential for coupling transport to ATP hydrolysis [13,41]; and (b) a relatively rigid region consisting of TM7–TM10, which is thought to serve as an anchor and provide structural stability during the catalytic cycle [42,43]. These structural distinctions suggest that sequence divergence in the mobile TM1–TM6 region may primarily affect energy transduction and ion access, whereas variations in the rigid TM7–TM10 region are more likely to modulate conformational stability and thermal tolerance, consistent with species-specific adaptations to environmental conditions.

### 4.2. Tissue Specificity of atp2a1

qRT-PCR analysis further delineated the tissue-specific expression of *atp2a1*, with highest levels in skeletal muscle, followed by testis, brain, heart, and gill. The strong expression in muscle is consistent with the well-established role of ATP2A1 in Ca^2+^ cycling during contraction and relaxation. Notably, studies on *atp2a1* expression patterns in teleosts remain limited, and, to our knowledge, no comprehensive analysis of its tissue-specific distribution has been reported to date. Similarly, our literature-based survey of crustacean SERCA homologs revealed a similar pattern, with the highest expression also detected in muscle tissues (Table S4), suggesting that the muscle-enriched expression of *atp2a1* may represent a conserved feature among aquatic animals. Interestingly, we observed a significant reduction in *atp2a1* expression in muscle under long-term cold acclimation, a phenomenon that warrants further investigation. In the brain, detectable expression agrees with reports of *atp2a1* expression during zebrafish development [4], suggesting a role in neuronal Ca^2+^ regulation. The relatively high signal in testis implies potential functions of ATP2A pumps in reproductive tissues. Mammalian studies have shown that ATP2A-sensitive Ca^2+^ stores are required for sperm function and the acrosome reaction, and pharmacological inhibition of ATP2A reduces sperm viability, supporting the involvement of ATP2A activity in spermatogenesis [44,45]. Although most evidence in mammals points to ATP2A2 as the major isoform in sperm, these observations motivate future cell type- and isoform-specific studies in fish to clarify whether *atp2a1* contributes to Ca^2+^ handling during spermatogenesis in *A. fasciatus*. The strong signal in gill is notable given that most work on branchial Ca^2+^ homeostasis emphasizes plasma-membrane transporters such as ECaC, PMCA, and NCX in ionocytes [46,47]; nonetheless, the unexpected high expression of *atp2a1* suggests that endoplasmic reticulum Ca^2+^ handling in gill cells may be more important than previously recognized, and this warrants further investigation in future studies.

### 4.3. Promoter Prediction and Transcriptional Regulation of atp2a1

Promoter analysis of *atp2a1* in *A. fasciatus* revealed the presence of multiple transcription factor binding motifs, whereas CpG island analysis indicated a lack of such islands in the promoter region. Since the promoter region of *atp2a1* in *A. fasciatus* lacks a CpG island, its muscle-specific transcription is likely governed predominantly by transcription factor binding rather than by CpG island–mediated epigenetic control [48]. Previous studies have suggested that ATF4 and CHOP may act as transcriptional regulators of *atp2a1*, particularly under endoplasmic reticulum stress conditions [49]. In addition, MyoD and Ebf3 have been implicated in activating *atp2a1* during muscle development, where Ebf3 directly binds the promoter and cooperates with MyoD to drive expression required for diaphragm function and muscle relaxation [50]. Promoter motif prediction identified KLF9, CTCF, MAZ, KLF5, ONECUT3, and HOXB13 as candidate regulators of *atp2a1*, thereby highlighting the potential mechanisms through which these factors may influence its transcription. KLF5 and KLF9 are Krüppel-like transcription factors that bind GC/GT-box motifs and regulate muscle and metabolic gene expression. KLF5 directly cooperates with MyoD and MEF2 to activate myogenic programs [51]. In contrast, KLF9 fine-tunes transcription under hormonal and metabolic regulation, acting indirectly through partners such as C/EBPα and PPARγ2 to modulate promoter responsiveness [52,53]. MAZ binds purine-rich GC motifs (e.g., GGGAGGG) and can either activate or repress transcription; its ability to interact with G-quadruplex structures [54]. In contrast, no direct experimental evidence currently links CTCF, ONECUT3, or HOXB13 to *atp2a1*.

These findings suggest that the regulation of *atp2a1* extends beyond its canonical role in calcium cycling, potentially linking it to growth and developmental processes. Evidence from comparative analyses of gene families further suggests that partial sequence divergence among homologous proteins can lead to functional divergence [18], raising the possibility that *atp2a1* in *A. fasciatus* has acquired species-specific adaptations related to muscle performance.

### 4.4. Thermal and Hydrodynamic Stress Response

The distinct transcriptional responses of *atp2a1* to environmental stimuli highlight its versatile role in physiological adaptation. Specifically, our data show that prolonged exposure to low temperature (16 °C) significantly reduces *atp2a1* expression in muscle, potentially reflecting a strategic downregulation of calcium cycling to match reduced metabolic demands in colder conditions. Previous studies have shown that thyroid hormone (TH) regulates Atp2a1 expression during cold exposure, which may promote its role in modulating thermogenesis [55]. Prolonged low-temperature conditions reduce overall metabolic activity, thereby decreasing *atp2a1* expression. In contrast, prior work in Japanese medaka under cold stress revealed increased *atp2a1* expression in skeletal muscle—particularly alongside elevated sarcolipin (Sln)—suggesting a role in non-shivering thermogenesis [9]. Similarly, a study on largemouth bass (*Micropterus salmoides*) found that *atp2a1* expression decreased under acute heat stress, suggesting that both thermal extremes can perturb calcium cycling in muscle cells. Together, these findings imply that *atp2a1* responds dynamically to temperature fluctuations, functioning as a key molecular component linking calcium homeostasis to muscle energy metabolism [56]. Moreover, increased flow velocity induces upregulation of *atp2a1*, implying a mechanosensitive regulatory mechanism enhancing muscle function under hydrodynamic stress. While direct investigations into flow-induced *atp2a1* expression in fish are limited, it aligns with broader observations that flow regimes influence muscle remodeling in zebrafish [57]. This mechanosensory responsiveness suggests that *atp2a1* may support enhanced contractility and cellular calcium homeostasis in dynamic aquatic environments. Together, these findings position *atp2a1* as a dynamic mediator of environmental responsiveness, integrating thermal and hydrodynamic cues to modulate muscle performance. This flexibility may be critical for *A. fasciatus* in habitats with fluctuating temperature and flow conditions.

## 5. Conclusions

This study presents a comprehensive characterization of the *atp2a1* gene in *A. fasciatus* using genomic, transcriptomic, and experimental approaches. Structural and phylogenetic analyses revealed strong conservation across teleosts with functional divergence from mammals in key domains. Tissue expression profiling demonstrated predominant transcription in skeletal muscle and reproductive organs, consistent with roles in Ca^2+^ regulation and muscle function. Promoter analysis identified several transcription factors binding motifs, suggesting transcriptional regulation independent of CpG island–mediated mechanisms. Importantly, long-term cold acclimation was associated with reduced *atp2a1* expression and lower SE inclusion, whereas moderate flow velocity showed the opposite trend, enhancing *atp2a1* transcription and promoting SE inclusion. Collectively, these findings indicate that *atp2a1* is not only central to Ca^2+^ cycling but may also contribute to growth regulation and environmental adaptation in teleosts, providing new insights into the molecular basis of physiological plasticity under fluctuating habitats.

## Figures and Tables

**Table 1:** *atp2a1* promoter prediction.

Start	End	Score	Promoter Sequence (5′−3′)
201	251	0.97	ggagggcactgtatatacacacacacacacacacacacacacacacacac
255	305	0.88	aaaaacatataataaaaaggagacaagtagatagaatagagaaagctagt
1038	1088	0.82	tcatataaattatatgaattcgccaaacgtagttataagttgtcacgaga
1480	1530	0.83	cagcactcctctaactgccccctcccatcagaggccacttggcccatcac
1921	1971	0.95	agggacaatcaataaaaggcacggatagacctgtttaactaagctttcgc

Note: Position 1 in the sequence corresponds to −2000 bp.

**Table 2:** Predicted transcription factor binding sites in the *atp2a1* promoter region.

Transcription Factor	Star/bp	End/bp	Strand	Score	q-Value	Matched Sequence
KLF9	216	226	+	10.4065	6.88 × 10^−5^	tacacacacac
	218	228	+	12.2033	2.57 × 10^−5^	cacacacacac
CTCF	1485	1517	+	12.2752	7 × 10^−6^	ctcctctaactgccccctcccatcagaggccac
MAZ	1498	1505	+	16.0816	8.13 × 10^−6^	cccctccc
KLF5	1497	1506	+	13.4082	2.25 × 10^−5^	ccccctccca
ONECUT3	1924	1935	+	13.3708	1.2 × 10^−5^	gacaatcaataa
HOXB13	1929	1937	+	13.0873	2.65 × 10^−5^	tcaataaaa

Note: Position 1 in the sequence corresponds to −2000 bp.

## Data Availability

The full-length transcripts raw data generated by PacBio sequencing platform has been deposited into the NGDC (National Genomics Data Center; https://ngdc.cncb.ac.cn/?lang=zh; accessed on 15 August 2025) with accession number PRJCA032910 and CRA020749. Long-term thermal acclimation sequence data are available under accession numbers PRJCA032913 and CRA020752, while long-term flow velocity acclimation sequence data are available under accession numbers PRJCA045242 and CRA029280.

## Supplementary Materials

The following supporting information can be downloaded at: https://www.mdpi.com/article/10.3390/genes16111385/s1, Figure S1: Comprehensive analysis of alternative splicing and polyadenylation events in the transcriptome. (A) Distribution of genes with more than one splicing isoform among the 14,947 genes analyzed. (B) KEGG pathway enrichment of genes exhibiting multiple splice isoforms, primarily associated with Human Diseases, Cellular Processes, and Environmental Information Processing. (C) Top 10% of genes with the highest frequency of exon skipping, including actin, *rad21*, *plk2*, *dcx*, *ccna2*, and *atp2a1*. (D) Genome-wide mapping of 16,547 polyadenylation (polyA) sites across 10,755 genes, highlighting 3743 genes with variable polyA sites. (E) KEGG pathway enrichment of genes with variable polyA sites, mainly linked to Human Diseases, Cellular Processes, Environmental Information Processing, and Organismal Systems. (F) Top 10% of genes with the highest number of polyA sites, including *atp2a1* and *mhy*. (G) Detailed characterization of *atp2a1* (AFCHR12_02800.1), which ranked among the top 10% in both splice isoform counts and polyA site number, revealing 781 isoforms, 3353 alternative splicing events, and eight distinct polyA sites; Figure S2: In silico prediction of structural features and transmembrane topology of Atp2a1. (A) Hydrophilicity and hydrophobicity profile of Atp2a1. (B) Protein domain structure of Atp2a1. (C) Predicted transmembrane domains of Atp2a1. (D) Signal peptide prediction of Atp2a1. (E) Secondary structure prediction of Atp2a1; Figure S3: CpG island prediction in the *atp2a1* promoter region. The analysis was performed with thresholds of window length > 100 bp, GC content > 50%, and observed/expected CpG ratio > 0.6, and no CpG islands were detected; Table S1: Tissue Sampling and Processing Methods for Female and Male *Acrossocheilus fasciatus* (1-year-old) in the Study of RNA Extraction, Sequencing and qRT-PCR; Table S2: Primer sequences of the target unigenes selected for analysis by qRT-PCR; Table S3: Amino acid homology comparison of Atp2a1 among *Acrossocheilus fasciatus* and other species; Table S4. Comparative overview of *atp2a1* tissue-specific expression patterns in crustacean species; Supplementary Material S1: Atp2a1 Nucleotide Sequence (cDNA, 5′→3′) and Predicted Amino Acid Sequence; Supplementary Material S2: Predicted 3D structure of the *Acrossocheilus fasciatus* Atp2a1 protein generated by SWISS-MODEL (PDB format); Supplementary Material S3: Predicted 3D structure of the *Onychostoma macrolepis* Atp2a1 protein generated by SWISS-MODEL (PDB format); Supplementary Material S4: 2 kb Upstream Regulatory Sequence of the *Acrossocheilus fasciatus* Gene AFchr12_02800 on Chromosome 12; Supplementary Material S5. Predicted transcription factor binding sites (TFBSs) in the *atp2a1* promoter; Supplementary Material S6: Alternative splicing information of *atp2a1* under different treatments, using 0 BL·s^−1^ and 24 °C as controls [58,59,60,61].

## Abbreviations

The following abbreviations are used in this manuscript:

AS	Alternative splicing
CBP	CREB-binding protein
CpG	Cytosine-phosphate-Guanine dinucleotide
DEG	Differentially expressed gene
ER	Endoplasmic reticulum
FDR	False discovery rate
FPKM	Fragments per kilobase of transcript per million mapped reads
GC	Guanine-Cytosine content
KEGG	Kyoto Encyclopedia of Genes and Genomes
MW	Molecular weight
NJ	Neighbor-joining (phylogenetic method)
ORF	Open reading frame
PCR	Polymerase chain reaction
pI	Isoelectric point
PSI	Percent spliced in (value for AS events)
qRT-PCR	Quantitative reverse transcription polymerase chain reaction
SE	Skipped exon
ATP2A1	Sarcoplasmic/endoplasmic reticulum calcium ATPase 1 ()
TMR	Transmembrane region
TF	Transcription factor
TFBS	Transcription factor binding site
TSS	Transcription start site
Note: The fully uppercase form (ATP2A1) refers to the mammalian ortholog; the capitalized form (Atp2a1) refers to the fish ortholog; and the lowercase italicized form (*atp2a1*) denotes the gene.	

## References

[1] Palmgren M.G., Nissen P. (2011). P-Type ATPases. Annu. Rev. Biophys..

[2] Periasamy M., Kalyanasundaram A. (2007). SERCA Pump Isoforms: Their Role in Calcium Transport and Disease. Muscle Nerve.

[3] Hirata H., Saint-Amant L., Waterbury J., Cui W., Zhou W., Li Q., Goldman D., Granato M., Kuwada J.Y. (2004). Accordion, a Zebrafish Behavioral Mutant, Has a Muscle Relaxation Defect Due to a Mutation in the ATPase Ca^2+^ Pump SERCA1. Development.

[4] Lai Y.-Y., Pai C.-W., Tsai I.-T., Chou C.-Y., Tsai C.-T., Chen Y.-H. (2011). Molecular Structure and Developmental Expression of Zebrafish *atp2a* Genes. Genes Genom..

[5] Toyoshima C., Nakasako M., Nomura H., Ogawa H. (2000). Crystal Structure of the Calcium Pump of Sarcoplasmic Reticulum at 2.6 Å Resolution. Nature.

[6] Arruda A.P., Nigro M., Oliveira G.M., de Meis L. (2007). Thermogenic Activity of Ca^2+^-ATPase from Skeletal Muscle Heavy Sarcoplasmic Reticulum: The Role of Ryanodine Ca^2+^ Channel. Biochim. Biophys. Acta Biomembr..

[7] Launikonis B.S., Murphy R.M. (2025). From Muscle-Based Nonshivering Thermogenesis to Malignant Hyperthermia in Mammals. Annu. Rev. Physiol..

[8] Xu H., Van Remmen H. (2021). The Sarco/Endoplasmic Reticulum Calcium ATPase (SERCA) Pump: A Potential Target for Intervention in Aging and Skeletal Muscle Pathologies. Skelet. Muscle.

[9] Robinson S., Hechter D., Almoumen F., Franck J.P. (2024). Sarcolipin (*sln*) and Sarcoplasmic Reticulum Calcium ATPase Pump (*serca1*) Expression Increase in Japanese Medaka (*Oryzias latipes*) Skeletal Muscle Tissue Following Cold Challenge. Comp. Biochem. Physiol. A Mol. Integr. Physiol..

[10] Robinson S., Wegner N.C., Sepulveda C.A., Franck J.P. (2024). Relative Sarcolipin (SLN) and Sarcoplasmic Reticulum Ca^2+^ ATPase (SERCA1) Transcript Levels in Closely Related Endothermic and Ectothermic Scombrid Fishes: Implications for Molecular Basis of Futile Calcium Cycle Non-Shivering Thermogenesis (NST). Comp. Biochem. Physiol. A Mol. Integr. Physiol..

[11] Chou M.-Y., Hsiao C.-D., Chen S.-C., Chen I.-W., Liu S.-T., Hwang P.-P. (2008). Effects of Hypothermia on Gene Expression in Zebrafish Gills: Upregulation in Differentiation and Function of Ionocytes as Compensatory Responses. J. Exp. Biol..

[12] Pan Y., Zvaritch E., Tupling A.R., Rice W.J., de Leon S., Rudnicki M., McKerlie C., Banwell B.L., MacLennan D.H. (2003). Targeted Disruption of the ATP2A1 Gene Encoding the Sarco (Endo)plasmic Reticulum Ca^2+^ ATPase Isoform 1 (SERCA1) Impairs Diaphragm Function and Is Lethal in Neonatal Mice. J. Biol. Chem..

[13] Zhao Y., Ogawa H., Yonekura S.-I., Mitsuhashi H., Mitsuhashi S., Nishino I., Toyoshima C., Ishiura S. (2015). Functional Analysis of SERCA1b, a Highly Expressed SERCA1 Variant in Myotonic Dystrophy Type 1 Muscle. Biochim. Biophys. Acta Mol. Basis Dis..

[14] Zhao B., Shi W., Guo Y., Chen Y., Wang H., He J., Chu Z. (2024). Effect of Temperature on the Growth, Feeding Performance, Gonadal Development, and Nutritive Compositions in the Muscle of Fry Stream Groupers, *Acrossocheilus fasciatus*. J. World Aquac. Soc..

[15] Guo Y., Dong C., Peng H., Zhang J., He J., Gao Y., Dai X., Zhao S., Chu Z., Zhao B. (2025). Behavioral Responses and Transcriptional Dynamics of the Stream Fish (*Acrossocheilus fasciatus*) under Temperature Change. Water Biol. Secur..

[16] He J., Wang H., Guo Y., Chu Z., Zhao B. (2022). Molecular Mechanism of Extreme Hypoxia Tolerance Difference between Male and Female Adult Fish and Juvenile Fish of *Acrossocheilus fasciatus* by Transcriptomics. Indian. J. Anim. Res..

[17] Huang J., Tong H., Gao B., Wu Y., Li W., Xiao P. (2024). Long-Term Exposure to Dimefluthrin Inhibits the Growth of *Acrossocheilus fasciatus*. Environ. Res..

[18] Guo Y., Wang S., Niyompano F., Li T., Chen J., Luo Z., Jiang X., Chen Y., Zhao B. (2025). Identification and Characterization of *hsp70* Gene Family in *Acrossocheilus fasciatus* Based on Genome and Full-Length Transcripts. Comp. Biochem. Physiol. D Genom. Proteom..

[19] Wei Z., Fang Y., Shi W., Chu Z., Zhao B. (2023). Transcriptional Modulation Reveals Physiological Responses to Temperature Adaptation in *Acrossocheilus fasciatus*. Int. J. Mol. Sci..

[20] Zheng J., Jiang J., Rui Q., Li F., Liu S., Cheng S., Chi M., Jiang W. (2024). Chromosome-Level Genome Assembly of *Acrossocheilus fasciatus* Using PacBio Sequencing and Hi-C Technology. Sci. Data.

[21] Yuan X., Tao L., Hu X., Lin R., Yang J., Feng M., Peng M., Liu W., Xiao Y. (2024). Expression Profile Analysis of Muscle Regulation Genes under Growth and Water Flow Stress in Zebrafish. Reprod. Breed..

[22] Gasteiger E., Hoogland C., Gattiker A., Duvaud S.E., Wilkins M.R., Appel R.D., Bairoch A., Walker J.M. (2005). Protein identification and analysis tools on the ExPASy server. The Proteomics Protocols Handbook.

[23] Thumuluri V., Almagro Armenteros J.J., Johansen A.R., Nielsen H., Winther O. (2022). DeepLoc 2.0: Multi-label subcellular localization prediction using protein language models. Nucleic Acids Res..

[24] Letunic I., Bork P. (2025). SMART v10: Three decades of the protein domain annotation resource. Nucleic Acids Res.

[25] Hallgren J., Tsirigos K.D., Pedersen M.D., Almagro Armenteros J.J., Marcatili P., Nielsen H., Winther O. (2022). DeepTMHMM predicts alpha and beta transmembrane proteins using deep neural networks. bioRxiv.

[26] Nielsen H., Teufel F., Brunak S., von Heijne G., Kihara D. (2024). SignalP: The evolution of a web server. Protein Bioinformatics.

[27] Buchan D.W., Moffat L., Lau A., Kandathil S.M., Jones D.T. (2024). Deep learning for the PSIPRED protein analysis workbench. Nucleic Acids Res..

[28] Waterhouse A., Bertoni M., Bienert S., Studer G., Tauriello G., Gumienny R., Heer F.T., de Beer T.A.P., Rempfer C., Bordoli L. (2018). SWISS-MODEL: Homology modelling of protein structures and complexes. Nucleic Acids Res..

[29] Fleming J., Magana P., Nair S., Tsenkov M., Bertoni D., Pidruchna I., Querino Lima Afonso M., Midlik A., Paramval U., Žídek A. (2025). AlphaFold Protein Structure Database and 3D-Beacons: New data and capabilities. J. Mol. Biol.

[30] Kumar S., Stecher G., Tamura K. (2016). MEGA7: Molecular evolutionary genetics analysis version 7.0 for bigger datasets. Mol. Biol. Evol..

[31] Madeira F., Madhusoodanan N., Lee J., Eusebi A., Niewielska A., Tivey A.R.N., Lopez R., Butcher S. (2024). The EMBL-EBI Job Dispatcher sequence analysis tools framework in 2024. Nucleic Acids Res..

[32] Lalitha S. (2000). Primer Premier 5. Biotech Softw. Internet Rep..

[33] Ren Y., Mu Y., Zhao B., Gao Y., Dai X., Chu Z. (2023). *dmrt3*, *nom1*, *abce1*, and *pkmyt1* Play Key Roles in Gonadal Sex Determination in *Acrossocheilus fasciatus*. Aquac. Int..

[34] Reese M.G. (2001). Application of a time-delay neural network to promoter annotation in the Drosophila melanogaster genome. Comput. Chem..

[35] Li L.C., Dahiya R. (2002). MethPrimer: Designing primers for methylation PCRs. Bioinformatics.

[36] Chen C., Wu Y., Li J., Wang X., Zeng Z., Xu J., Liu Y., Feng J., Chen H., He Y. (2023). TBtools-II: A “one for all, all for one” bioinformatics platform for biological big-data mining. Mol. Plant.

[37] Love M.I., Huber W., Anders S. (2014). Moderated estimation of fold change and dispersion for RNA-seq data with DESeq2. Genome Biol..

[38] Shen S., Park J.W., Lu Z.-X., Lin L., Henry M.D., Wu Y.N., Zhou Q., Xing Y. (2014). rMATS: Robust and Flexible Detection of Differential Alternative Splicing from Replicate RNA-Seq Data. Proc. Natl. Acad. Sci. USA.

[39] Zhao B., Huang H., Guo Y., Uzanyinema T., Yu J., Zhang Q., Chu Z. (2025). Morphological and transcriptomic analysis of testes in juvenile *Acrossocheilus fasciatus* under different water flow conditions. Oceanol. Limnol. Sin..

[40] Raguimova O.N., Smolin N., Blackwell D., Bovo E., Zima A., Robia S. (2017). A Discrete Loop of the SERCA N-Domain Interacts with Phospholamban and Stabilizes a Compact Conformation of the SERCA Cytosolic Headpiece. Biophys. J..

[41] Fernández-de Gortari E., Aguayo-Ortiz R., Autry J.M., Espinoza-Fonseca L.M. (2020). A Hallmark of Phospholamban Functional Divergence Is Located in the N-terminal Phosphorylation Domain. Comput. Struct. Biotechnol. J..

[42] Olesen C., Picard M., Winther A.-M.L., Gyrup C., Morth J.P., Oxvig C., Møller J.V., Nissen P. (2007). The Structural Basis of Calcium Transport by the Calcium Pump. Nature.

[43] Toyoshima C. (2009). How Ca^2+^-ATPase Pumps Ions across the Sarcoplasmic Reticulum Membrane. Biochim. Biophys. Acta Mol. Cell Res..

[44] Garriga F., Martínez-Hernández J., Parra-Balaguer A., Llavanera M., Yeste M. (2025). The Sarcoplasmic/Endoplasmic Reticulum Ca^2+^-ATPase (SERCA) Is Present in Pig Sperm and Modulates Their Physiology over Liquid Preservation. Sci. Rep..

[45] Lawson C., Dorval V., Goupil S., Leclerc P. (2007). Identification and Localisation of SERCA 2 Isoforms in Mammalian Sperm. Mol. Hum. Reprod..

[46] Liao B.-K., Deng A.-N., Chen S.-C., Chou M.-Y., Hwang P.-P. (2007). Expression and Water Calcium Dependence of Calcium Transporter Isoforms in Zebrafish Gill Mitochondrion-Rich Cells. BMC Genom..

[47] Pinto P.I., Matsumura H., Thorne M.A., Power D.M., Terauchi R., Reinhardt R., Canário A.V.M. (2010). Gill Transcriptome Response to Changes in Environmental Calcium in the Green Spotted Puffer Fish. BMC Genom..

[48] Salem M., Al-Tobasei R., Ali A., Kenney B. (2022). Integrated Analyses of DNA Methylation and Gene Expression of Rainbow Trout Muscle under Variable Ploidy and Muscle Atrophy Conditions. Genes.

[49] Chami M., Oulès B., Szabadkai G., Tacine R., Rizzuto R., Paterlini-Bréchot P. (2008). Role of SERCA1 Truncated Isoform in the Proapoptotic Calcium Transfer from ER to Mitochondria during ER Stress. Mol. Cell.

[50] Jin S., Kim J., Willert T., Klein-Rodewald T., Garcia-Dominguez M., Mosqueira M., Fink R., Esposito I., Hofbauer L.C., Charnay P. (2014). Ebf Factors and MyoD Cooperate to Regulate Muscle Relaxation via Atp2a1. Nat. Commun..

[51] Hayashi S., Manabe I., Suzuki Y., Relaix F., Oishi Y. (2016). Klf5 regulates muscle differentiation by directly targeting muscle-specific genes in cooperation with MyoD in mice. eLife.

[52] Pei H., Yao Y., Yang Y., Liao K., Wu J.R. (2011). Krüppel-like factor KLF9 regulates PPARγ transactivation at the middle stage of adipogenesis. Cell Death Differ..

[53] Gans I.M., Grendler J., Babich R., Jayasundara N., Coffman J.A. (2021). Glucocorticoid-responsive transcription factor Krüppel-like factor 9 regulates *fkbp5* and metabolism. Front. Cell Dev. Biol..

[54] Bossone S.A., Asselin C., Patel A.J., Marcu K.B. (1992). MAZ, a zinc finger protein, binds to c-MYC and C2 gene sequences regulating transcriptional initiation and termination. Proc. Natl. Acad. Sci. USA.

[55] Little A.G., Seebacher F. (2013). Thyroid hormone regulates muscle function during cold acclimation in zebrafish (*Danio rerio*). J. Exp. Biol..

[56] Zhao X., Wang Y., Wang Z., Luo T., Huang J., Shao J. (2024). Analysis of Differential Alternative Splicing in Largemouth Bass after High Temperature Exposure. Animals.

[57] Suriyampola P.S., Zúñiga-Vega J.J., Jayasundara N., Flores J., Lopez M., Bhat A., Martins E.P. (2023). River Zebrafish Combine Behavioral Plasticity and Generalized Morphology with Specialized Sensory and Metabolic Physiology to Survive in a Challenging Environment. Sci. Rep..

[58] Yu J., Feng W., Chen X., Song C., Su S., Ge J., Tang Y. (2022). Molecular Cloning and Functional Characterization of Sarco/Endoplasmic Reticulum Ca^2+^-ATPase from Chinese Mitten Crab (*Eriocheir sinensis*). Aquac. Res..

[59] Mandal A., Arunachalam S.C., Meleshkevitch E.A., Mandal P.K., Boudko D.Y., Ahearn G.A. (2009). Cloning of Sarco-Endoplasmic Reticulum Ca^2+^-ATPase (SERCA) from Caribbean Spiny Lobster *Panulirus argus*. J. Comp. Physiol. B.

[60] Wang Y., Luo P., Zhang L., Hu C., Ren C., Xia J. (2013). Cloning of Sarco/Endoplasmic Reticulum Ca^2+^-ATPase (SERCA) Gene from White Shrimp *Litopenaeus vannamei* and Its Expression Level Analysis under Salinity Stress. Mol. Biol. Rep..

[61] Roegner M.E., Chen H.Y., Watson R.D. (2018). Molecular Cloning and Characterization of a Sarco/Endoplasmic Reticulum Ca^2+^-ATPase (SERCA) from Y-Organs of the Blue Crab Callinectes sapidus. Gene.

