# Supplementary material for: Structural Conservation and Transcriptional Plasticity of atp2a1 in Acrossocheilus fasciatus Under Temperature and Flow Acclimation
Source: Genes (Basel). 2025 Nov 15;16(11):1385. doi: 10.3390/genes16111385 (PMC12652649; doi:10.3390/genes16111385)
Supplement: Supplementary file 1 [file genes-16-01385-s001.zip › genes-3910373-supplementary figures and tables.pdf]

Table S1. Tissue sampling and processing methods for female and male *Acrossocheilus fasciatus* (1-year-old) in the study of RNA extraction, sequencing and qRT-PCR.

Tissue Sample	Female Sample Handling	Male Sample Handling	Processing Method	qRT-PCR
Brain	Mixed	Mixed	Combined processing for all samples	✔
Gill	Mixed	Mixed	Combined processing for all samples	✔
Heart	Mixed	Mixed	Combined processing for all samples	✔
Spleen	Mixed	Mixed	Combined processing for all samples	✔
Head Kidney	Mixed	Mixed	Combined processing for all samples	✔
Liver	Mixed	Mixed	Combined processing for all samples	✔
Intestine	Mixed	Mixed	Combined processing for all samples	✔
Skin	Mixed	Mixed	Combined processing for all samples	✔
Dorsal Muscle	Mixed	Mixed	Combined processing for all samples	✔
Testes	None	Separate processing	Separate processing for male samples	✔
Ovaries	Separate processing	None	Separate processing for female samples	✔

Table S2. Primer sequences of the target unigenes selected for analysis by qRT-PCR.

Primer name	Sequence (5' - 3')	Number
*βac*t-F	CCCAGAATCCTATTGTTACCC	XM_026238990.1
βact-R	CCTCGCATACATAGTGCCATT	XM_026238990.1
atp2a1-F	GGATCCGTGGCGCTGTCTAC	AFchr12_02800.1
atp2a1-R	AGCCCAGGGTCTCCACAGAG	AFchr12_02800.1

Table S3. Amino acid homology comparison of Atp2a1 among *Acrossocheilus fasciatus* and other species.

	1	2	3	4	5	6	7	8	9	10
Mus musculus	100	96.68	83.75	84.36	84.56	84.46	84.16	84.36	84.96	84.66
Homo sapiens	96.68	100	83.85	84.36	84.36	84.56	84.36	84.56	84.86	84.76
Danio rerio	83.75	83.85	100	94.05	93.84	93.84	94.65	94.45	94.65	94.45
Acrossocheilus fasciatus	84.36	84.36	94.05	100	98.08	95.86	96.17	96.06	96.37	96.17
Onychostoma macrolepis	84.56	84.36	93.84	98.08	100	95.86	96.06	96.06	96.67	95.86
Cyprinus carpio	84.46	84.56	93.84	95.86	95.86	100	96.17	96.17	96.68	96.57
Labeo rohita	84.16	84.36	94.65	96.17	96.06	96.17	100	96.17	96.78	96.77
Sinocyclocheilus grahami	84.36	84.56	94.45	96.06	96.06	96.17	96.17	100	96.97	96.77
Puntigrus tetrazona	84.96	84.86	94.65	96.37	96.67	96.68	96.78	96.97	100	97.58
Carassius auratus	84.66	84.76	94.45	96.17	95.86	96.57	96.77	96.77	97.58	100

Table S4. Comparative overview of *atp2a1* tissue-specific expression patterns in crustacean species.

Species	Gills	Heart	Liver /hepatopancreas	Intestine	Muscle	Eyestalks	Y-organs	Green gland/	Reference
Eriocheir sinensis	+		+	+	+++	+	+		[1]
Panulirus argus	+	++		+	+++			+	[2]
Homarus americanus		++	+	+	+++			+	[2]
Litopenaeus vannamei	+		+++		+				[3]
Callinectes sapidus	+		+++		+++		+		[4]

Note: "+", "++", and "+++" indicate relative expression levels of *atp2a1* (SERCA1) in the corresponding tissues, based on qualitative or semi-quantitative evidence reported in the cited studies. "+" = low expression; "++" = moderate expression; "+++" = high expression; blank cells indicate that no expression data were available or not reported in the reference.

Reference

[1] Yu, J.; Feng, W.; Chen, X.; Song, C.; Su, S.; Ge, J.; Tang, Y. Molecular Cloning and Functional Characterization of Sarco/Endoplasmic Reticulum Ca^{2+}-ATPase from Chinese Mitten Crab (*Eriocheir sinensis*). *Aquac. Res* 2022, 53(13), 4676–4688.

[2] Mandal, A.; Arunachalam, S.C.; Meleshkevitch, E.A.; Mandal, P.K.; Boudko, D.Y.; Ahearn, G.A. Cloning of Sarco-Endoplasmic Reticulum Ca^{2+}-ATPase (SERCA) from Caribbean Spiny Lobster *Panulirus argus*. *J. Comp. Physiol. B* 2009, 179(2), 205–214.

[3] Wang, Y.; Luo, P.; Zhang, L.; Hu, C.; Ren, C.; Xia, J. Cloning of Sarco/Endoplasmic Reticulum Ca^{2+}-ATPase (SERCA) Gene from White Shrimp *Litopenaeus vannamei* and Its Expression Level Analysis under Salinity Stress. *Mol. Biol. Rep* 2013, 40(11), 6213–6221.

[4] Roegner, M.E.; Chen, H.Y.; Watson, R.D. Molecular Cloning and Characterization of a Sarco/Endoplasmic Reticulum Ca^{2+}-ATPase (SERCA) from Y-Organs of the Blue Crab Callinectes sapidus. *Gene* 2018, 673, 12–21.

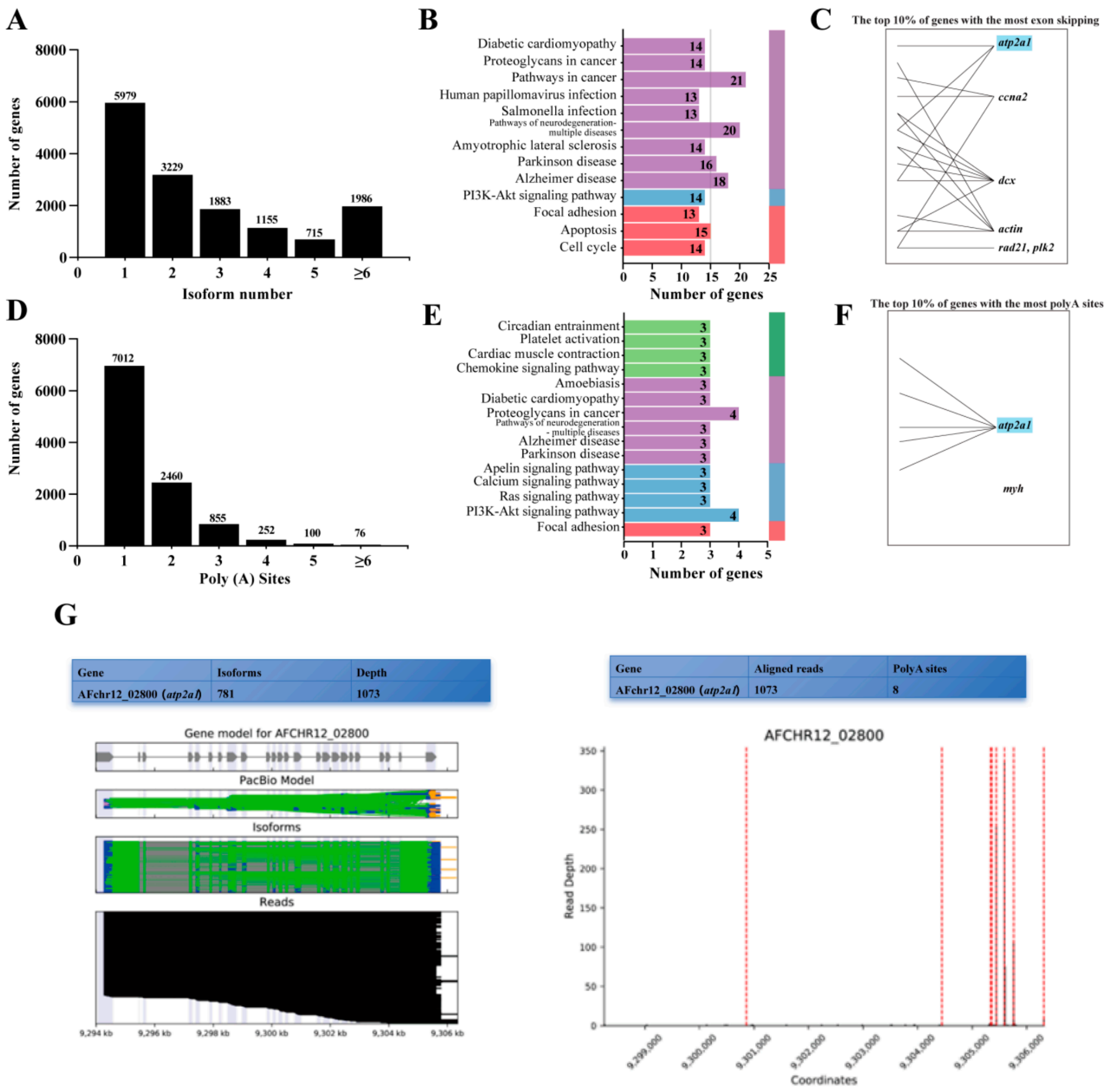

Figure S1. Comprehensive analysis of alternative splicing and polyadenylation events in the transcriptome. (A) Distribution of genes with more than one splicing isoform among the 14,947 genes analyzed. (B) KEGG pathway enrichment of genes exhibiting multiple splice isoforms, primarily associated with Human Diseases, Cellular Processes, and Environmental Information Processing. (C) Top 10% of genes with the highest frequency of exon skipping, including *actin*, *rad21*, *plk2*, *dcx*, *ccna2*, and *atp2a1*. (D) Genome-wide mapping of 16,547 polyadenylation (polyA) sites across 10,755 genes, highlighting 3,743 genes with variable polyA sites. (E) KEGG pathway enrichment of genes with variable polyA sites, mainly linked to Human Diseases, Cellular Processes, Environmental Information Processing, and Organismal Systems. (F) Top 10% of genes with the highest number of polyA sites, including *atp2a1* and *mhy*. (G) Detailed characterization of *atp2a1* (AFCHR12_02800.1), which ranked among the top 10% in both splice isoform count and polyA site number, revealing 781 isoforms, 3,353 alternative splicing events, and eight distinct polyA sites.

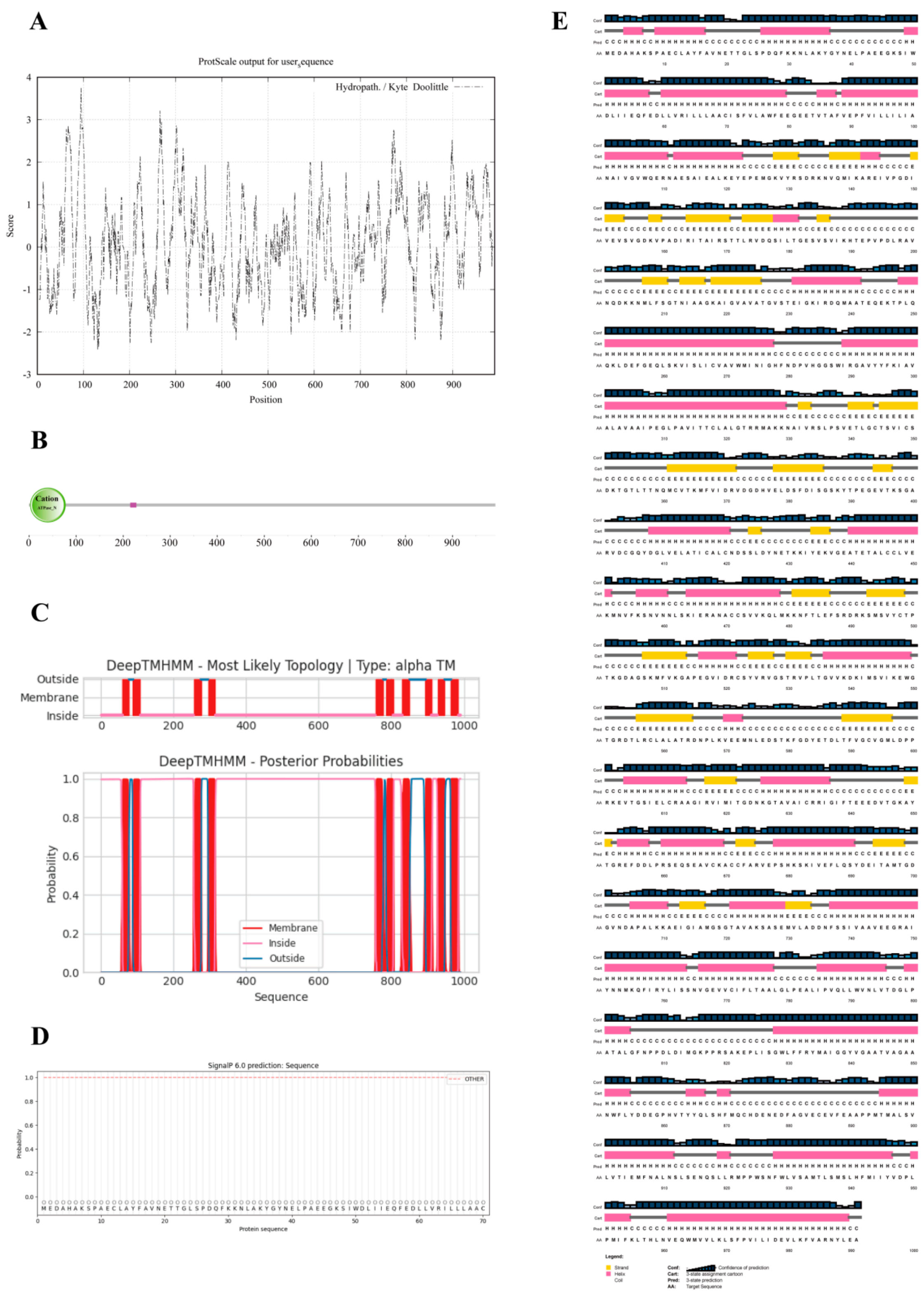

Figure S2. In silico prediction of structural features and transmembrane topology of Atp2a1. (A) Hydrophilicity and hydrophobicity profile of Atp2a1. (B) Protein domain structure of Atp2a1. (C) Predicted transmembrane domains of Atp2a1. (D) Signal peptide prediction of Atp2a1. (E) Secondary structure prediction of Atp2a1.

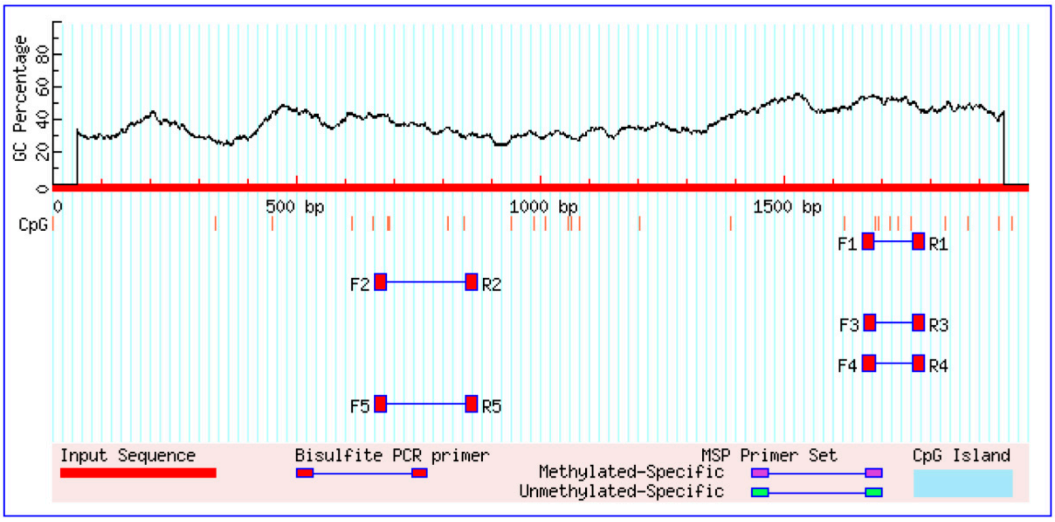

Sequence Name:
Sequence Length: 2000

CpG island prediction results
(Criteria used: Island size > 100, GC Percent > 50.0, Obs/Exp > 0.6)
No CpG islands were found in your sequence

Figure S3. CpG island prediction in the *atp2a1* promoter region. The analysis was performed with thresholds of window length > 100 bp, GC content > 50%, and observed/expected CpG ratio > 0.6, and no CpG islands were detected.
