# Supplementary material for: Structural Conservation and Transcriptional Plasticity of atp2a1 in Acrossocheilus fasciatus Under Temperature and Flow Acclimation
Source: Genes (Basel). 2025 Nov 15;16(11):1385. doi: 10.3390/genes16111385 (PMC12652649; doi:10.3390/genes16111385)
Supplement: Supplementary file 1 [file genes-16-01385-s001.zip › Supplementary Material S1.pdf]

>*Acrossocheilus fasciatus atp2a1*　（AFchr12_02800.1）

ATGGAGGATGCCCATGCCAAGTCCCCGGCCGAATGTCTGGCCTACTTCGCAGTTAATGA
AACCACAGGTCTTTCCCCGGATCAGTTCAAGAAGAACCTGGCTAAGTACGGCTACAAT
GAGCTGCCAGCTGAGGAGGGAAAATCCATCTGGGACCTGATCATTGAGCAGTTTGAAG
ATCTGTTGGTCAGAATTTTGCTGCTTGCTGCCTGCATCTCTTTCGTCCTGGCCTGGTTTG
AGGAAGGTGAAGAGACTGTCACTGCCTTTGTTGAGCCTTTTGTCATTTTGCTCATTCTC
ATTGCCAATGCCATCGTCGGTGTGTGGCAGGAGCGTAATGCTGAGAGCGCCATTGAGG
CTCTGAAGGAGTATGAGCCTGAGATGGGCAAGGTCTACCGTTCTGACAGAAAGAACG
TCCAGATGATCAAGGCCAGAGAGATTGTCCCTGGTGACATTGTGGAGGTGTCTGTTGG
TGATAAAGTTCCTGCTGACATCAGGATTACTGCTATCCGTTCCACCACCCTTCGTGTTG
ACCAGTCCATCCTGACTGGTGAGTCCGTCAGTGTGATCAAGCACACCGAGCCTGTCCC
CGACCTCAGAGCCGTCAATCAGGACAAAAAGAACATGCTTTTCTCTGGCACAAACATT
GCTGCTGGCAAGGCTATTGGCGTTGCTGTCGCTACTGGTGTATCCACTGAGATTGGTAA
GATCCGTGACCAGATGGCTGCCACAGAGCAGGAGAAGACCCCTCTGCAGCAGAAACT
GGATGAGTTTGGTGAGCAGCTCTCTAAGGTTATCTCTCTGATCTGCGTCGCTGTCTGGA
TGATCAACATCGGCCACTTCAATGACCCCGTCCATGGTGGATCCTGGATCCGTGGCGCT
GTCTACTACTTCAAGATCGCTGTTGCTCTGGCTGTAGCTGCTATCCCTGAGGGTCTGCC
TGCTGTCATCACTACCTGTCTGGCTCTTGGTACCAGACGTATGGCCAAGAAAAATGCCA
TTGTCCGTTCACTGCCCTCTGTGGAGACCCTGGGCTGTACTTCTGTCATCTGCTCCGAC
AAGACTGGCACCCTGACCACCAATCAGATGTGTGTGACCAAAATGTTCGTCATTGATA
GAGTTGATGGTGATCACGTTGAACTTGACTCCTTTGATATCTCTGGCTCCAAGTACACA
CCCGAGGGTGAGGTCACAAAGTCTGGTGCTCGTGTTGACTGCGGTCAGTATGACGGTT
TAGTTGAGTTGGCCACCATCTGCGCCCTCTGCAACGATTCCTCCCTTGACTACAATGAG
ACCAAGAAGATCTATGAGAAGGTCGGTGAGGCCACTGAAACTGCTTTGTGCTGCTTGG
TTGAGAAGATGAATGTTTTTAAGAGCAATGTTAACAACCTGTCCAAGATTGAGAGAGC
AAATGCCTGCTGTAGTGTTGTGAAGCAGCTAATGAAGAAGAACTTCACTCTGGAGTTC
TCCCGTGACAGGAAATCCATGTCTGTGTACTGTACCCCTACCAAGGGTGATGCAGGCA
GCAAAATGTTTGTGAAGGGCGCTCCAGAGGGTGTGATTGACAGGTGTTCCTATGTACG
TGTTGGTTCTACTCGGGTACCCCTGACTGGTGTTGTAAAAGATAAGATCATGTCCGTCA
TCAAGGAGTGGGGTACTGGCCGTGACACTCTGCGTTGCCTGGCACTTGCCACCAGAG
ACAATCCCCTGAAGGTTGAAGAAATGAACCTTGAGGACTCTACTAAATTTGGTGACTAT
GAGACTGACTTGACCTTCGTTGGCTGTGTCGGTATGTTGGATCCCCCCCGTAAAGAAG
TTACTGGCTCCATTGAACTGTGCAGGGCTGCTGGCATTCGTGTTATCATGATCACTGGT
GACAACAAGGGCACTGCTGTGGCCATCTGCCGTCGTATTGGCATCTTTACTGAGGAGG
AGGATGTAACTGGCAAGGCTTACACCGGCCGAGAGTTTGATGACCTGCCCCGTAGTGA
ACAGAGCGAAGCTGTCTGTAAGGCATGCTGCTTTGCCCGTGTTGAGCCCTCCCACAAG
TCTAAGATTGTTGAGTTCCTGCAGAGCTACGATGAGATTACTGCTATGACTGGTGATGG
TGTCAACGATGCCCCTGCCTTGAAGAAGGCAGAAATTGGCATTGCCATGGGCTCTGGC
ACTGCCGTTGCCAAGTCAGCCTCTGAGATGGTCCTGGCCGATGACAACTTCTCTTCTAT
TGTGGCTGCCGTTGAGGAAGGCAGAGCCATTTACAACAACATGAAGCAGTTTATCCGT
TACCTGATTTCTTCCAATGTTGGGGAGGTCGTCTGTATTTTCCTGACTGCTGCTCTTGGT
CTGCCTGAGGCCCTGATCCCAGTCCAACTGCTGTGGGTGAACTTGGTGACTGATGGTC
TGCCCGCCACCGCCCTGGGCTTCAACCCCCCTGATCTTGATATCATGGGCAAGCCTCCT
CGCTCTGCCAAAGAGCCCCTGATCTCTGGCTGGCTGTTCTTCAGATACATGGCCATTGG

TGGTTATGTGGGTGCTGCTACTGTGGCTGGTGCTGCCAACTGGTTCCTGTATGATGATG
AGGGTCCTCATGTCACCTACTATCAGCTGTCTCACTTCATGCAGTGCCATGACGAGAAC
GAGGACTTCGCTGGCGTTGAGTGTGAGGTGTTTGAGGCTGCTCCACCCATGACCATGG
CCCTGTCTGTCTTGGTCACAATTGAGATGTTCAACGCTCTCAACAGCTTGTCTGAGAAT
CAGTCCTTGTTGCGCATGCCTCCATGGAGCAATTTCTGGCTAGTGTCTGCCATGACCCT
CTCCATGTCCCTCCACTTCATGATCATCTATGTGGACCCCCTGCCCATGATTTTCAAACT
AACTCACTTGAACGTGGAACAGTGGATGGTGGTACTGAAGCTTTCTTTCCCCGTTATCC
TCATTGATGAggtgctgaagtttgttgcccGCAACTACCTGGAGGCCTAA

>*Acrossocheilus fasciatus atp2a1* ORF1（AFchr12_02800.1）
MEDAHAKSPAECLAYFAVNETTGLSPDQFKKNLAKYGYNELPAEEGKSIWDLIIEQFEDLL
VRILLLAACISFVLAWFEEGEETVTAFVEPFVILLILIANAIVGVWQERNAESAIEALKEYEP
EMGKVYRSDRKNVQMIKAREIVPGDIVEVSVGDKVPADIRITAIRSTTLRVDQSILTGESVS
VIKHTEPVPDLRAVNQDKKNMLFSGTNIAAGKAIGVAVATGVSTEIGKIRDQMAATEQEKT
PLQQKLDEFGEQLSKVISLICVAVWMINIGHFNDPVHGGSWIRGAVYYFKIAVALAVAAIPE
GLPAVITTCLALGTRRMAKKNAIVRSLPSVETLGCTSVICSDKTGTLTTNQMCVTKMFVID
RVDGDHVELDSFDISGSKYTPEGEVTKSGARVDCGQYDGLVELATICALCNDSSLDYNET
KKIYEKVGEATETALCCLVEKMNVFKSNVNNLSKIERANACCSVVKQLMKKNFTLEFSRD
RKSMSVYCTPTKGDAGSKMFVKGAPEGVIDRCSYVRVGSTRVPLTGVVKDKIMSVIKEW
GTGRDTLRCLALATRDNPLKVEEMNLEDSTKFGDYETDLTFVGCVGMLDPPRKEVTGSIE
LCRAAGIRVIMITGDNKGTAVAICRRIGIFTEEEDVTGKAYTGREFDDLPRSEQSEAVCKAC
CFARVEPSHKSKIVEFLQSYDEITAMTGDGVNDAPALKKAEIGIAMGSGTAVAKSASEMVL
ADDNFSSIVAAVEEGRAIYNNMKQFIRYLISSNVGEVVCIFLTAALGLPEALIPVQLLWVNL
VTDGLPATALGFNPPDLDIMGKPPRSAKEPLISGWLFFRYMAIGGYVGAATVAGAANWFL
YDDEGPHVTYYQLSHFMQCHDENEDFAGVECEVFEAAPPMTMALSVLVTIEMFNALNSL
SENQSLLRMPPWSNFWLVSAMTLSMSLHFMIIYVDPLPMIFKLTHLNVEQWMVVLKLSFP
VILIDEVLKFVARNYLEA
