# Supplementary material for: Structural Conservation and Transcriptional Plasticity of atp2a1 in Acrossocheilus fasciatus Under Temperature and Flow Acclimation
Source: Genes (Basel). 2025 Nov 15;16(11):1385. doi: 10.3390/genes16111385 (PMC12652649; doi:10.3390/genes16111385)
Supplement: Supplementary file 1 [file genes-16-01385-s001.zip › Supplementary Material S4.pdf]

>cds.AFchr12_02800.1+ Up_Stream_Len 2000 (5'−3')

tgggtgccaatatgtgaccaacatgaactaaagaaagcatttatttcacaatgagattttccccccactttcaattgttttactttaatgaaaggttag
aattttgtgaattttctgaatgaaagctcaaaaggataaacaatgcagatttattttcacagccacctttgctcatatttaccaagagtgccaatatta
gtggagggcactgtatatacacacacacacacacacacacacacacacacacacacaaaaacatataataaaaaggagacaagtagataga
atagagaaagctagtgttagatgcctttttttataaagaaaacaaacgaatagagagtgcaagtgtttttttttaagaaagaaaacaaatagataaa
atggaattagaatagagagtgctaaagttagatggtcaactaaagatggaagagatgtgttttagccgtttcttgaacatggcttgggactcagct
gcatgaattgagctgggcaggtcattccaccaggagggaacagttaatgtaaaagtttgtgaaagtgatttttttgcctctttgggatggcacaata
attccttcccaatacaataaaatgtctcacttgatcacgtgtgatcagatcactgaaaaccaggtggaaactgggcctgacgaatccttcactga
atgattttaatcagcgtaacgtctcattattttcagtaatgcccctcttgagcaaatcctttgttaaaaacaacaatgctgtaggagacactgtgctta
ctaaattatggagagctttgagaacttttaatctctaaacgttttctgtgtaaaataaacacaaataactgtttcgaagggcagccagacaaaaaca
atgaaatgataataatgaaatgtctattccaatctcatgagaaacataactattttataatgtagcaatttcgtaacaatttgtacaatgtgattcaaaa
agtaagaataaagtcagtggcgcataaccagatggtaagaaaacgtacaaatgagactatatatgaattcatataaattatatgaattcgccaaa
cgtagttataagttgtcacgagagtgtgttagacatgaaatactgtaaatatgttttcagtaattagccatggatatttggctaaaatgcctagtgat
gctctacatcattttctagttttttaatcagatcttaatgctacgacaggtctgaaaaggatcatacactctcagaaataaaggtacatgtctgtacct
aaagagtccattttggtaccttaaaggtacatattagaacctaataggtacaaaagtataccttttgaaaacagtgacagcttttgtacctttatttct
gagagtgaaggaaatgattgtgttcttgtccacgccaaaccaatgtgtggatgtcagtgtgacactttaagcctcccatgtaaccacactcctcta
atgcttgttccaaattaaccttccagcactcctctaactgccccctcccatcagaggccacttggcccatcactcactctagctctctcaccttctc
cactctccttattccactgaccatgcatgttccaaaaacagatcaacttttcatttcagtgctaccccctacgtatgctactgcctcatgctccttgcc
caccaaataacccccacatcctcaagtctgagatgccgaagacgttcaacccctagactctaactcggccctgcaaagccatacgcaactgat
tggaattttggggtgtggcgacaggcaactttgaccaatagggcagctggcagtagtataaaacactgcacatgtgtgaatttattcgctctttct
gtcagtccatcacttctgtgaggagggggtccaagcaacgggggcagattttatagtacagagagcaacaagagcaatagggacaatcaa
taaaaggcacggatagacctgtttaactaagctttcgccactcttgaagatccttcacccttgacagg

Note: Position 1 in the sequence corresponds to –2000 bp. Green indicates predicted potential transcription start sites (TSSs).
